# Supplementary material for: Veteran trees have divergent effects on beetle diversity and wood decomposition
Source: PLoS One. 2021 Mar 18;16(3):e0248756. doi: 10.1371/journal.pone.0248756 (PMC7971458; doi:10.1371/journal.pone.0248756)
Supplement: S1 Fig — Values on top right are the Pearson’s correlation coefficient. (DOCX) [file pone.0248756.s001.docx]

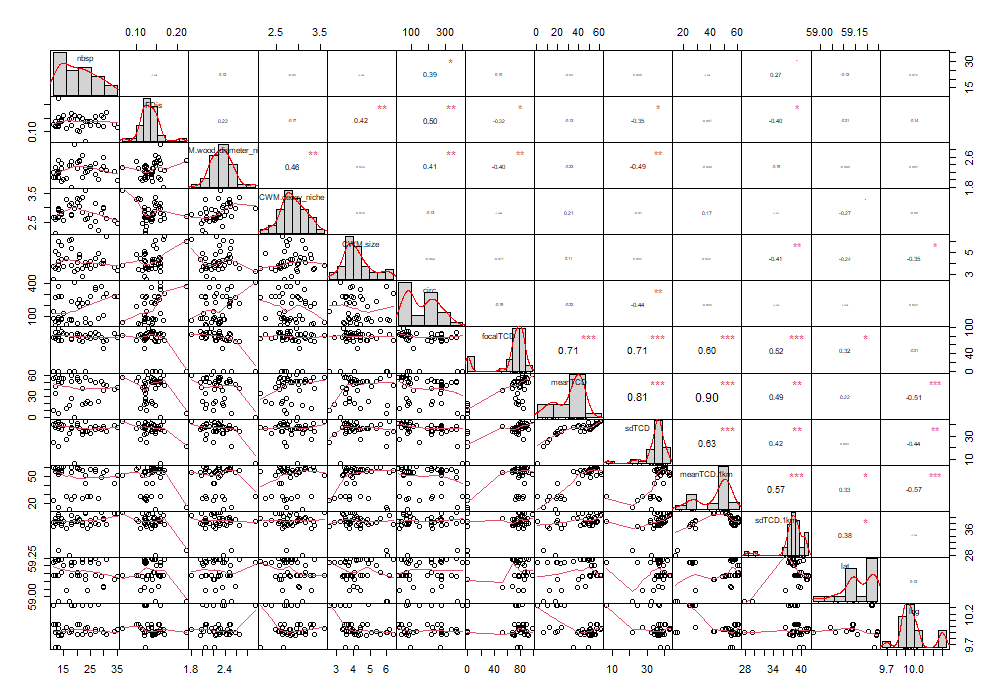


**S1 Fig:** Correlation matrix with continuous variables used in the analysis of beetle diversity and wood decomposition rates around oaks in Southern Norway. Values on top right are the Pearson's correlation coefficient.
