# Supplementary material for: Veteran trees have divergent effects on beetle diversity and wood decomposition
Source: PLoS One. 2021 Mar 18;16(3):e0248756. doi: 10.1371/journal.pone.0248756 (PMC7971458; doi:10.1371/journal.pone.0248756)
Supplement: S2 Fig — Histograms show beetle species richness, density of wood bundles, functional diversity (FDis), community weighted mean (CWM) of species’ wood decay stage preference, species’ wood diameter preference, and beetle body length. (DOCX) [file pone.0248756.s002.docx]

**S2
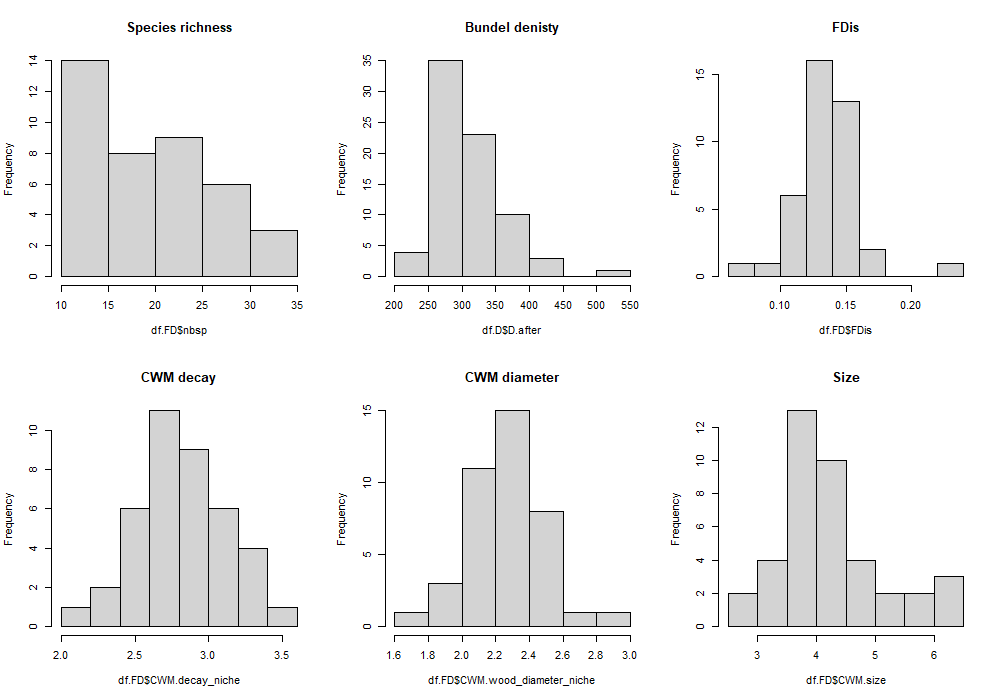
 Fig:** Distribution of values from beetle sampling and experimentally added wood bundles around oaks in southern Norway. Histograms show beetle species richness, density of wood bundles, functional diversity (FDis), community weighted mean (CWM) of species’ wood decay stage preference, species’ wood diameter preference, and beetle body length.
