# Supplementary material for: Veteran trees have divergent effects on beetle diversity and wood decomposition
Source: PLoS One. 2021 Mar 18;16(3):e0248756. doi: 10.1371/journal.pone.0248756 (PMC7971458; doi:10.1371/journal.pone.0248756)
Supplement: S1 Table — Beetles were classified as being wood decomposers based on being involved in primary or secondary wood decomposition at any point in their life stages. This included the following feeding types (FT): xylophagous (x), mycetophagous (m), saprophagous (s) and polyphagous (p). Additionally, the trait data regarding the beetle’s body length (mm), wood diameter preference (WD pref) and wood decay stage preference (D pref), and references for their feeding type is provided. (DOCX) [file pone.0248756.s003.docx]

**S1 Table:** Beetle species captured in flight intercept traps in veteran (VT) and young (YT) trees and characterized as wood-decomposers. Beetles were classified as being wood decomposers based on being involved in primary or secondary wood decomposition at any point in their life stages. This included the following feeding types (FT): xylophagous (x), mycetophagous (m), saprophagous (s) and polyphagous (p). Additionally, there is trait data regarding the beetle’s body length (mm), wood diameter preference (WD pref) and wood decay stage preference (D pref), and references for their feeding type.
