# Supplementary material for: Veteran trees have divergent effects on beetle diversity and wood decomposition
Source: PLoS One. 2021 Mar 18;16(3):e0248756. doi: 10.1371/journal.pone.0248756 (PMC7971458; doi:10.1371/journal.pone.0248756)
Supplement: S2 Table — All models compare values between veteran and young oak trees. All response variables were modeled with LMMs with Gaussian distribution. (DOCX) [file pone.0248756.s004.docx]

**S2 Table:** Estimated regression parameters, standard errors and P‐values from models that predicted the starting wet weight and density of the bundles, bundle wetness after the experiment, and tree cover density (TCD) at 20m and 100m scales. All models compare values are veteran and young oak trees. All response variables were modeled with LMM with Gaussian distribution.

| **Response variable and predictors** | | **Estimate** | **Standard error** | **P value** |
| --- | --- | --- | --- | --- |
| **Bundle start weight** |  |  |  |  |
| intercept |  | 1866.08 | 47.45 | <0.001 |
| Type of tree | (veteran) | 25.86 | 47.15 | 0.585 |
| placement | (hanging) | -57.39 | 47.15 | 0.228 |
|  |  |  |  |  |
| **Bundle start density** |  |  |  |  |
| intercept |  | 0.5644666 | 0.0169000 | <0.001 |
| Type of tree | (veteran) | -0.0001062 | 0.0184897 | 0.995 |
| placement | (hanging) | -0.0024019 | 0.0185061 | 0.897 |
|  |  |  |  |  |
| **Bundle wetness** |  |  |  |  |
| intercept |  | 179.616 | 6.050 | <0.001 |
| Type of tree | (veteran) | 8.280 | 5.911 | 0.166 |
| placement | (hanging) | -6.714 | 5.910 | 0.260 |
|  |  |  |  |  |
| **TCD 20m** |  |  |  |  |
| intercept |  | 13.258 | 3.794 | <0.001 |
| Type of tree | (veteran) | -3.794 | 5.366 | 0.484 |
| Surroundings | (forest) | 29.801 | 2.915 | <0.001 |
|  |  |  |  |  |
| **TCD 100m** |  |  |  |  |
| intercept |  | 31.404 | 2.0058 | <0.001 |
| Type of tree | (veteran) | 0.1734 | 2.5179 | 0.945 |
| Surroundings | (forest) | 25.1818 | 2.915 | <0.001 |
|  |  |  |  |  |
|  |  |  |  |  |
|  |  |  |  |  |
|  |  |  |  |  |
